# Merged Group Tractography Evaluation with Selective Automated Group Integrated Tractography

**DOI:** 10.3389/fnana.2016.00096

**Published:** 2016-10-13

**Authors:** David Q. Chen, Jidan Zhong, David J. Hayes, Brendan Behan, Matthew Walker, Peter S.-P. Hung, Mojgan Hodaie

**Affiliations:** ^1^Institute of Medical Science, Faculty of Medicine, University of Toronto, TorontoON, Canada; ^2^Krembil Research Institute, University Health Network, TorontoON, Canada; ^3^Division of Neurosurgery, Toronto Western Hospital and University of Toronto, TorontoON, Canada; ^4^Joint Department of Medical Imaging, University Health Network, TorontoON, Canada

**Keywords:** tractography, group-wise tractography, merged tractography, diffusion imaging, pipeline, multi-tensor, HARDI

## Abstract

**Introduction:** Tractography analysis in group-based studies across large populations has been difficult to implement. We propose Selective Automated Group Integrated Tractography (SAGIT), an automated group tractography software platform that incorporates multiple diffusion magnetic resonance imaging (dMRI) practices which will allow great accessibility to group-wise dMRI. We use a merged tractography approach that permits evaluation of tractography datasets at the group level. We also introduce an image normalized overlap score (NOS) that measures the quality of the group tractography results. We deploy SAGIT to evaluate deterministic and probabilistic constrained spherical deconvolution (CST*_det_*, CST*_prob_*) tractography, eXtended Streamline Tractography (XST), and diffusion tensor tractography (DTT) in their ability to delineate different neuroanatomy, as well as validating NOS across these different brain regions.

**Materials and methods:** Magnetic resonance sequences were acquired from 42 healthy adults. Anatomical and group registrations were performed using Automated Normalization Tools. Cortical segmentation was performed using FreeSurfer. Four tractography algorithms were used to delineate six sets of neuroanatomy: fornix, facial/vestibular-cochlear cranial nerve complex, vagus nerve, rubral–cerebellar decussation, optic radiation, and auditory radiation. The tracts were generated both with and without region of interest filters. The generated visual reports were then evaluated by five neuroscientists.

**Results:** At a group level, merged tractography demonstrated that different methods have different fiber distribution characteristics. CST*_prob_* is prone to false-positives, and thereby suitable in anatomy with strong priors. CST*_det_* and XST are more conservative, but have greater difficulty resolving hemispherical decussation and distant crossing projections. DTT consistently shows the worst reproducibility across the anatomies. Linear regression of rater scores against NOS shows significant (*p* < 0.05) correlation of the two sets of scores in filtered tractography. However, correlations are not significant (*p* > 0.05) for unfiltered tractography.

**Conclusion:** The tractography results demonstrated reliable and consistent performance of SAGIT across multiple subjects and techniques. Through SAGIT, we quantifiably demonstrated that different algorithms showed different strengths and weaknesses at a group level. While no single algorithm seems to be suitable for all anatomical tasks, it is useful to consider the use of a mix of algorithms for different anatomical segments. SAGIT appears to be a promising group-wise tractography analysis approach for this purpose.

## Introduction

Diffusion magnetic resonance imaging (dMRI) tractography is an imaging analysis technique that permits non-invasive visualization of white matter anatomy *in vivo* (Basser et al., 2000). It is based on the observation that the Brownian motion of water molecules within white matter fibers is constrained by the axonal bundles, and therefore such anisotropic water diffusion can be used to probe tissue microstructure (Bihan et al., 2001). By scanning in multiple angular directions using diffusion weighted imaging (DWI) sequence, a model of diffusion can be estimated for each voxel of the scanned image volume.

In the classic single-tensor dMRI (also known as diffusion tensor image; DTI) model (Basser and Jones, 2002), a single tensor is constructed at each voxel based on Gaussian model of diffusion, which describes the dominant diffusion direction. Using the tensor information, tractography algorithms (Mori and van Zijl, 2002) can be used to trace out structures within the DTI volume. The limitation of DTI is that there is insufficient information to resolve areas with crossing fibers with one tensor per voxel. Improvements over the limits of the DTI model, particularly in increasing angular resolution to improve crossing fibers resolution has been a subject of great interest (Tuch et al., 2002; Fritzsche et al., 2010; Jeurissen et al., 2012).

To improve the crossing fiber information per voxel, significant changes in DWI acquisition strategies are required. These methods include diffusion spectrum imaging (DSI; Wedeen et al., 2005, 2008) which samples the Q-space in a Cartesian grid, and Q-ball imaging that is based on the Funk–Radon transform (Descoteaux et al., 2007; Cho et al., 2008) that samples the Q-space in a spherical shell. These approaches, as well as more complex multi-shell sampling strategies, aim to construct and sharpen an orientation distribution function (ODF) in order to provide better approximate of the underlying diffusion. These methods require complex and long DWI acquisitions that are often unsuitable for clinical applications. Alternatively, methods based on spherical deconvolution (SD) can be used on high angular resolution diffusion imaging (HARDI) scans of lesser angular resolution. The approach is to model the HARDI signal as a convolution of the fiber orientations. The resulting deconvoluted fiber orientation distribution (FOD) shows better discrimination of fiber directions compared to Q-ball under similar scanning parameters, but is susceptible to image noise (Tournier et al., 2004; Anderson, 2005; Dell’Acqua et al., 2007). Alternatively, multi-tensor model-based approaches estimate the fiber configuration with the assumption that there are no more than two or three crossings in a given voxel. These include eXtended Streamline Tractography (XST), which can delineate lateral projections of the corticospinal tract in the motor cortex, in DWI scans with 50 gradient directions (Qazi et al., 2009). Other tractography algorithm include stochastically resolving tract propagation based on established white matter prior probability (probabilistic tractography; Behrens et al., 2007). Probabilistic approaches, however, are predominantly image-based, and result in a visitation volume image that needs to be visualized volumetrically.

There is increasing recognition in the field on the value of incorporating dMRI tractography into group-based studies in large populations. However, group tractography analysis has a number of challenges. The dMRI-derived geometries are difficult to register due to the large dimensionality of variables and low spatial resolution. Direct linear and non-linear deformations of either the tensor/ODF field can also confound diffusion metrics. Inter-subject anatomical variability also poses challenges for direct tractography geometric clustering and registration, making tract bundle identifications difficult. Specific anatomical selection also needs to be considered. TRACULA (TRActs Constrained by Underlying Anatomy) (Yendiki et al., 2011), for example, is able to perform group tractography analysis on pre-defined major white matter bundles based on ball-and-stick probabilistic tractography template, however, there are no solutions available for fully customizable anatomy that incorporates new advances in dMRI tractography. Recently there are efforts to evaluate tractography algorithms with synthetic datasets such as Tractometer (Côté et al., 2012, 2013), as well as attempts to generate synthetic data from individuals (Wilkins et al., 2015). From these comparative studies, there is evidence that no single tractography algorithm is superior for the reconstruction of all white matter tracts. There are no detailed studies that have attempted to compare tractography algorithms across larger populations to determine their relative suitability for different white matter tract identification.

In the clinical setting, the anatomy of interest is often specific, and needs to be put in a context of high individual variability, therefore manual targeting of regions of interest (ROIs) are unavoidable. Across a population, manual ROI delineation is at the risk of high ROI placement variations due to operator bias. The complexity of tractography data across a population increases dramatically when iterative tuning of tractography parameters is considered. Therefore, a combined and automated approach to group tractography in neuroimaging is highly desirable.

There is a lack of well-organized diffusion tractography software framework that bridges the gap between group image registration, diffusion image processing, large-scale tractography delineations, and tractography evaluation. We propose an automated tractography software platform that incorporates existing and proven dMRI techniques, in order for group-wise dMRI to be more accessible to researchers. Selective Automated Group Integrated Tractography (SAGIT^1^) is a configurable and fully automated tractography generation and comparison pipeline. Its key contributions are: (a) automated preprocessing, registration and tractography across an arbitrary number of subjects; (b) flexibility in ROI definitions that allows high customizability for anatomical targeting; (c) expressive ROI query that works with pre-defined segmentation masks such as FreeSurfer; (d) compatibility with a number of popular tractography software that allows parameter iterations for consistent results. SAGIT enables researchers to examine the result of different tractography algorithms at the group level. We also introduce an image-based score (normalized overlap score, NOS) that can quantify the quality of the group tractography results across different tractography algorithms to further assist decision making for researchers.

Finally, we deploy our system to the evaluation of various tractography methods in their ability to delineate neuroanatomy that are highly specific but difficult to image. These include supra and infratentorial structures such as small white matter bundles in heavy crossing fibers (cranial nerves), pontine decussation (rubrocerebellar pathway), and curved central pathways (fornix, visual, and auditory radiations). We recruited five neuroanatomists to evaluate and rate the resulting visual reports generated by SAGIT. We then compared the results of NOSs and the human raters to assess the automated NOS as a tractography reproducibility metric.

## Materials and Methods

### MRI Acquisitions

Magnetic resonance (MR) images were acquired using GE Signa HDx 3 Tesla scanner with an eight-channel head-coil. MR sequences were acquired from 42 healthy adults (mean age 30.4 ± 8.1 years). Ethics approval was granted by the University Health Network Research Ethics Board (Toronto, Canada), MR images were acquired at the Toronto Western Hospital, and all subjects gave their informed written consent. DWI were acquired with 1 B0 scan, 60 gradient directions, 3 mm slice thickness and in-plane resolution of 0.9375 × 0.9375 mm, *b* = 1000 s/mm^2^, echo time (TE) = 86.4 ms, repetition time (TR) = 17,000 ms, flip angle = 90°, field of view (FOV) = 240 mm, and matrix = 256 × 256. T1 fast spoiled gradient echo (FSPGR) anatomical scans were acquired with 1 mm slice thickness and in-plane resolution of 0.9375 × 0.9375 mm, slice spacing = 1 mm, TE = 5.052 ms, TR = 11.956 ms, flip angle = 20°, FOV = 240 mm, and matrix = 256 × 256.

### Preprocessing

**FIGURE 1:**
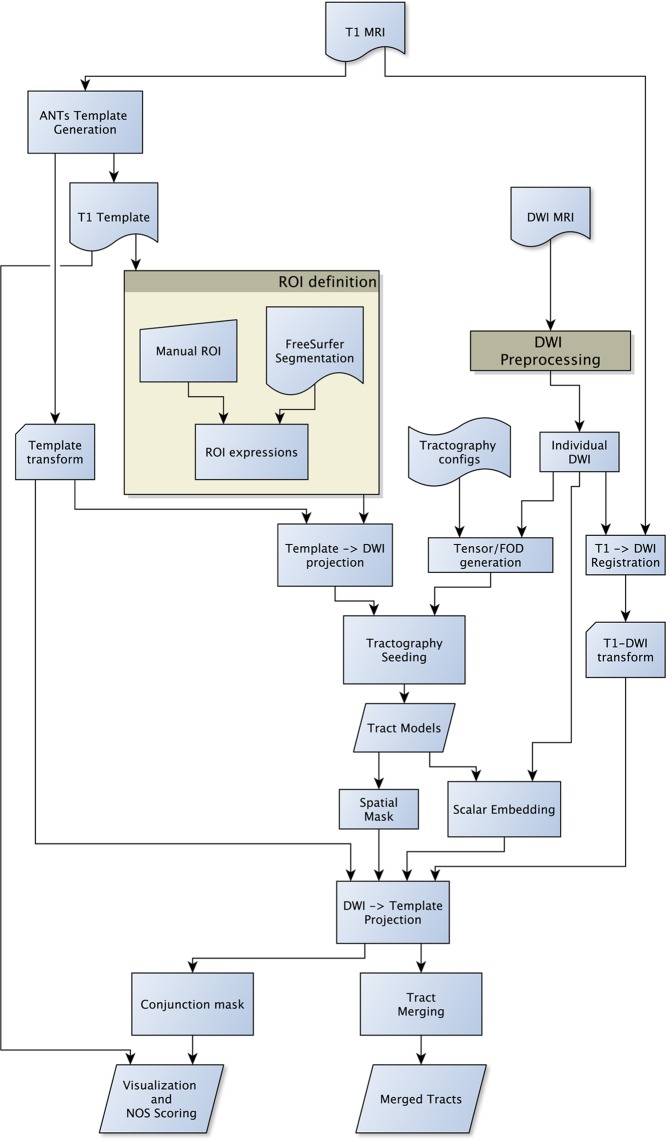
**Flow diagram of the SAGIT group tractography framework.** The SAGIT framework is designed to be fully configurable and extensible.

In order to automate the generation of tractography using multiple methods and minimize user-bias, the SAGIT framework is created (**Figure 1**). DWI sequences were corrected for eddy-current and motion distortions with appropriate rotational corrections to gradient vectors (Leemans and Jones, 2009). Fractional anisotropy (FA), axial diffusivity (AD), and radial diffusivity (RD) maps were generated from the DWI. A group-specific average anatomical template was created with subject T1 image, and intra-subject T1 to DWI space was obtained using symmetric diffeomorphic registration (SyN) with Automated Normalization Tools (ANTs; Avants et al., 2008). Cortical and subcortical segmentations based on the T1 images were obtained with FreeSurfer segmentation software (Fischl et al., 2002).

### Tractography Delineation

The tractography methods attempted to reconstruct the bilateral white matter anatomy of the fornix, facial/vestibular-cochlear cranial nerve complex (CN VII/VIII), vagus nerve (CN X), red nucleus pontine decussation (RN), lateral geniculate visual pathway (LGN) and medial geniculate auditory pathway (MGN). Seeding ROIs were defined on the group template, and projected to the individual DWI space.

Tractography filters were defined using a custom query expression to generate gray matter/white matter boundary inclusion and exclusion filter masks based on the FreeSurfer segmentations (see **Supplementary Material Data Sheet 1**). No filtering rules were applied for the fornix. The fornix is used as a control group to judge rating bias. The unfiltered (filtering rules were not applied) versions of each anatomy were also generated for evaluation.

Single-tensor diffusion tensor tractography (DTT) using 3D Slicer version 3 (Tuch et al., 2000; Pieper et al., 2006), two-tensor tractography using XST (Qazi et al., 2009), constrained SD-based deterministic streamline tractography (CST*_det_*; Tournier et al., 2012) and constrained SD-based probabilistic tractography (CST*_prob_*; Tournier et al., 2010) using MRtrix version 3 were evaluated (see **Supplementary Material Data Sheet 2** for parameters).

FA, AD, and RD scalars were sampled and embedded into the tractography models using tri-linear interpolation from the corresponding image volumes in native DWI space. The affine and non-linear image registration transforms were applied to deform the native tracts from the DWI space to the template space; all corresponding tracts of the same anatomy in template space were then merged into one single tractography model, to obtain the merged group tractography geometry.

The individual tractography models were also converted to binary spatial images, and the resulting images were transformed to the template space. Multiple binary images were stacked together to form the conjunction percentage overlap image. The overlaps were then visualized using concentric isosurfaces created from step-wise (10%) thresholds of the underlying overlap volume using the MayaVI data visualization library (Ramachandran and Varoquaux, 2011). A color lookup table with even visual brightness falloff (van der Walt and Smith, 2015) was chosen to avoid visual judgment biases from the color presentation. Different viewpoints (axial, coronal, sagittal, and perspective) were created for each visualization, and then composed together to create the visual report.

### Tractography Evaluation

**FIGURE 2:**
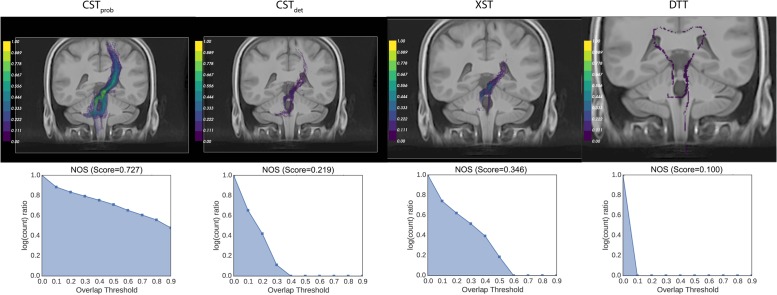
**Examples of NOSs of different conjunction images: the figure shows the coronal view of the left red nucleus tractography projections, as produced by different algorithms.** The NOS correlates closely with the visual color scale. It can also resolve visually similar comparisons, such as the rating between XST and CST*_det_* results.

The generated visual reports were then evaluated by five neuroscientists in a blinded study using a set of rating criteria (see **Supplementary Material Table 1**). The conjunction images were at the same time used to generate NOSs. The NOS quantified the conjunction image generated by overlapping tractography masks to meaningfully determine the spatial agreement independent of anatomy. The assumption was that given a conjunction image, where its voxel value *s* denotes a range of overlap percentages between 0 and 100%, and value *1* denotes the 100% overlap value, we formalize it as s ∈ [0,1], and define NOS as:

$$n^{-1}\overset{n-1}{\underset{i=0}{\Sigma }}\frac{In\left(\nu_{i}\right)}{In\left(\nu_{0}\right)}$$

where *n* was the number of bins; *v*_0_ was the number of voxels where *s >* 0; *v*_i_ was the number of voxels with $0<s\geq \frac{i}{n}$; this study used *n* = 10. Examples of NOS behavior are available in **Figure 2** for the left red nucleus projections.

The rater scores were then normalized to each anatomy, and the normalized rater scores were linearly regressed against the NOSs for correlations. Linear regression of rater scores against NOS was performed using the R-statistics library (R Development Core Team, 2012).

## Results

**FIGURE 3:**
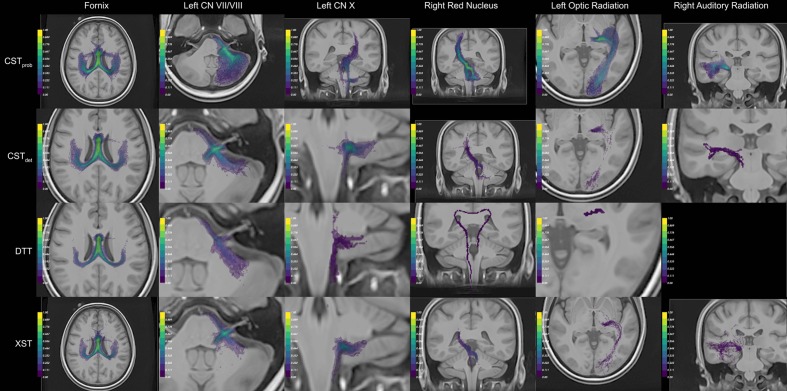
**Example of the auto-generated visual panels.** The panels that best represent the resulting anatomy are compiled. For anatomical reference, the best representative anatomy image slice is composed with the tract conjunction map. The color scale of the conjunction image represents the percentage reproducible for each region, where 1 = 100%. For each algorithm (rows), different visual perspectives are created. Together six different anatomies were delineated (columns). To see the full visual reports generated, please see **Supplementary Material Data Sheet 3**.

**FIGURE 4:**
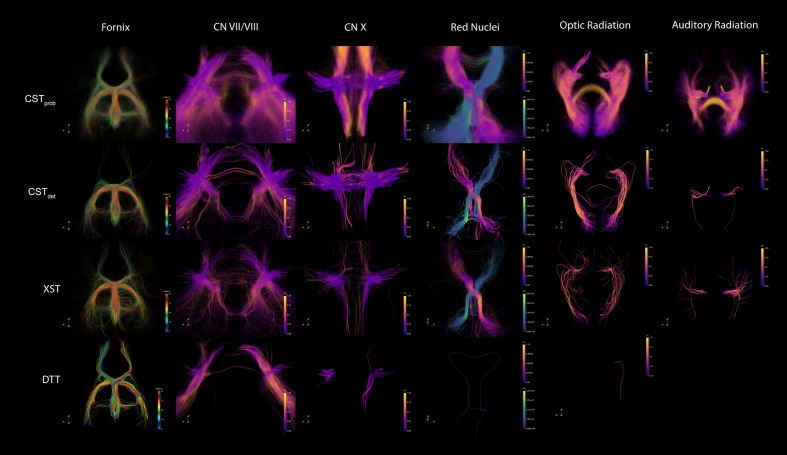
**Example of merged tractography models for each of the techniques and anatomies.** The fornix is colored to distinguish tracts from each of the 42 subjects, in order to demonstrate the effect of combining the group data. The other anatomies are colored by FA intensity. The specific FA measures from each point were sampled from their native DWI space before tractography deformation. The grouping of the similar FA measures across the subjects show that the registrations were accurate. The red nuclei show different metrics (FA and AD) depending on lateralization, in order to highlight the pontine decussation.

**FIGURE 5:**
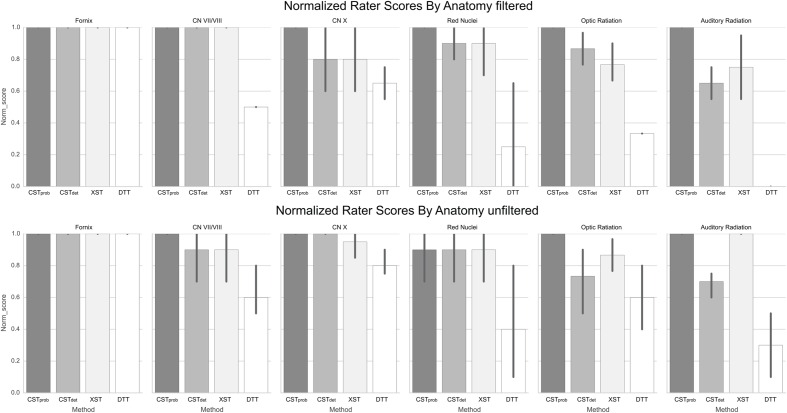
**Normalized anatomical scores as rated by experts.** The error bars denote standard deviations of the ratings. The fornix serves as the control anatomy, therefore the represented filtered and unfiltered image are identical. It can be observed that unfiltered anatomy increases rater variability in some and decrease in others.

**FIGURE 6:**
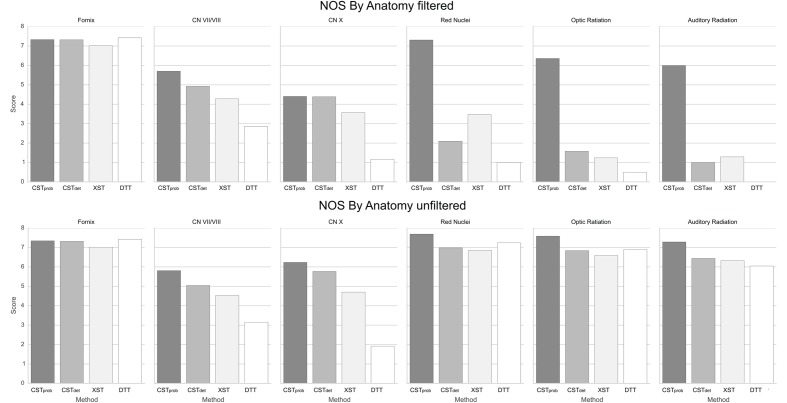
**NOSs for each anatomy.** It can be noted that having ROI filters severely reduces reproducibility in deterministic methods.

The fornix was shown to be consistent across the different algorithms. The visual report (**Figure 3**; the complete visual report can be found in **Supplementary Material Data Sheet 3**) suggested that the subregions of the fornix were consistently delineated. The merged fornix tracts (**Figure 4**) showed different patterns of streamline distributions. CST*_prob_* and XST both showed wide spread streamline dispersions, while CST*_det_* and particularly DTT were limited to the region of the fornix. The fornix ratings (**Figure 5**) showed essentially no variability between raters. The fornix NOSs (**Figure 6**) similarly were consistent across the algorithms with little variability. Both filtered and unfiltered ratings were identical.

For the CN VII/VIII, CST*_prob_*, CST*_det_*, and XST were all able to delineate the cranial as well as brainstem segments of the nerve (**Figure 3**). CST*_prob_*, however, also delineated much of the cerebellum. DTT by Slicer was not able to delineate the brainstem portions of the fiber and therefore was scored lower than the other methods. These differences in delineation can be more clearly seen in the merged tracts (**Figure 4**), where the XST also showed further lateral delineations of CN VII/VIII. In unfiltered tractography, there was much more inter-rater variability for all except CST*_prob_* (**Figure 5**). CST*_det_* and XST also showed lower average rating when unfiltered. The NOS showed similar trend where DTT resulted in the lowest score (**Figure 6**). The NOS also showed higher score for CST*_det_* when comparing to XST. There were no notable changes in NOSs in unfiltered tractography.

For CN X, CST*_prob_* was rated the highest with no inter-rater variability. CST*_det_* and XST resulted in more rating variability and a lower average score (**Figure 5**). CST*_prob_* visually showed delineations that extended into the ipsilateral higher brain regions even with filters (**Figures 3** and **4**). DTT showed the worst reproducibility, although its anatomy was recognizable in a small number of individuals. In unfiltered tractography, CST*_det_* and XST showed reduced variability, and visually showed delineations of ascending and brainstem projects. DTT also showed improved ratings when unfiltered (**Figure 5**). NOSs (**Figure 6**) for both filtered and unfiltered CN X tractography similarly showed an overall increase in score with unfiltered tractography.

For the red nucleus projections, visually CST*_prob_* showed the most consistent reproducibility of the decussation (**Figures 3** and **4**), with XST showing higher reproducibility over CST*_det_*. DTT failed to delineate the pathway and instead delineated an erroneous path that decussated at the corpus callosum. CST*_prob_*, CST*_det_*, and XST resulted similar ratings, with unfiltered tracts showing lower rating and higher variability. DTT had the lowest rating, and very high variability (**Figure 5**). NOS of the red nucleus projections highlighted the stronger performance of the CST*_prob_* over the other algorithms, and XST result was scored higher than CST*_det_* (**Figure 6**). The NOSs of unfiltered tracts were notably higher for all algorithms, as the resulting tracts seemed to delineate much of the cortical spinal projections.

The optic radiation showed the greatest reproducibility with CST*_prob_*, where the Meyer’s loop could be seen in the result (**Figures 3** and **4**). CST*_det_* and XST showed low reproducibility of less than 20%, and DTT failed to delineate any structure on the right side (see **Supplementary Material Data Sheet 3**). The ratings reflected the observation (**Figure 5**), with CST*_prob_* having the full rating. CST*_det_* was rated higher in filtered tractography, while XST was rated higher when unfiltered. DTT also showed higher ratings when unfiltered. NOS characterized the drop-off in reproducibility in filtered tractography for the deterministic methods (**Figure 6**), and the trend of rating under filtered tractography matched well with the ratings. NOS for unfiltered tractography, however, showed little match with ratings.

The auditory radiation was the least reproducible region. In filtered tractography, although CST*_prob_* showed high reproducibility in tractography (**Figures 3** and **4**), it was not clear if its delineation was correct. Visually there were wide spread area of false positives toward the occipital and anterior temporal lobe. The deterministic methods all showed poor ability to reach the Heschl’s gyrus. DTT was completely unable to produce any structures at all. For filtered ratings, CST*_prob_* was the highest, and with XST rated higher than CST*_det_* (**Figure 5**). DTT scored 0 due to the lack of delineated structures. When unfiltered, all algorithms showed widespread false positives in the hemispheres. Unfiltered ratings were over all higher than filtered ratings. NOSs trends for filtered tractography matched well with ratings, but also highlighted the large differences in reproducibility (**Figure 6**). While NOSs under unfiltered tractography did not correlate with ratings.

**FIGURE 7:**
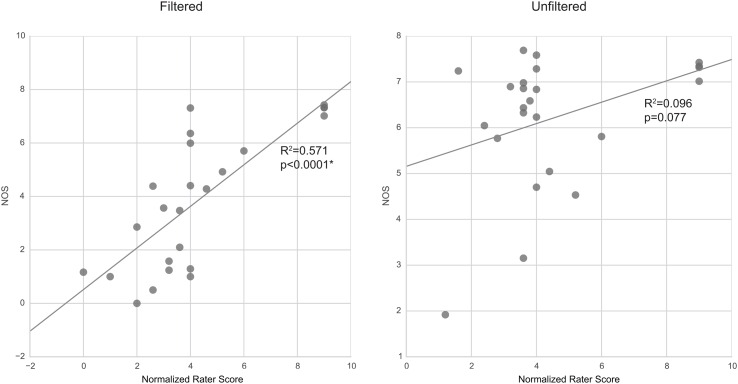
**Correlation between rating scores and NOS.** Filtered tractography NOS is significantly (*p* < 0.05) correlated with ratings. However, unfiltered tractography show no significance.

Linear regression of rater scores against NOS (**Figure 7**) showed significant (*p* < 0.05) correlation of the two sets of scores in filtered tractography. However, correlations were not significant (*p* > 0.05) for unfiltered tractography. It can be observed that NOS in unfiltered tractography agrees well with visual intuition, however, rater variability and anatomical reports suggested greater false positives when presented with unfiltered results.

## Discussion

The present study performed automated group tractography generation and collation report on six sets of neuroanatomy in 42 subjects using four different types of tractography algorithms. The proposed automation pipeline performed with great reliability. We have designed the automated pipeline to offer the ability to generate and collate tractography on a large scale. The key benefit of this approach is the vastly improved inter-subject tract reproducibility as a result of ROI filtering, and speed for tractography data generation. The method offers flexibility for researchers in ROI placements and parameter control for these tractography algorithms. The merged tractography permits additional analysis of the group-based anatomical reconstructions, and the auto-generated visual reports also reduce data confusion and improve research efficiency.

The most critical component of the pipeline is the reliability of image registrations. For this purpose, we picked ANTs since it is state-of-the-art and widely tested and deployed in numerous neuroimaging studies (Klein et al., 2009; Avants et al., 2011). For T1 to DWI co-registration, we used mean DWI (MDWI) as the registration intermediate. MDWI appears to be a reliable T1–DWI co-registration intermediate in the absence of reverse-blip DWI acquisitions (Chen et al., 2015a). Future studies can also potentially improve T1–DWI co-registration, for example, with the possible use of anisotropic power image (Dell’Acqua et al., 2014).

We have deployed the pipeline to the task of delineating six sets of neuroanatomy from clinical DWI data. The anatomies were chosen to test the limits of the SAGIT pipeline as well as the tractography algorithms. These anatomies are often shown in an existing atlas, and contain curving projections that are difficult to delineate, but are nevertheless well defined and contain clear anatomical landmarks for ease of judgment. In total, we included two sets of cranial nerves (CN VII/VIII and CN X) for their small but precise anatomy; rubrocerebellar projections of the red nucleus, due to the difficulty of imaging the pontine decussation; optical radiation for its well defined landmarks and the Meyer’s loop, which is difficult to image; auditory radiation, which is particularly difficult due to its course, that passes through a three-way crossing in temporal lobe. The fornix serves as the control anatomical structure. We have previously studied fornix subregion anatomies extensively (Chen et al., 2015b), and it is a popular anatomy to showcase the ability of new tractography algorithms due to the curvature of the forniceal crura (Garyfallidis et al., 2014).

It can be observed that results of averaged tractography image conjunction across a group are visually similar to probabilistic tractography in an individual, and that the group junction image seems to stabilize as the number of subject increase. It can be argued that the conjunction image captures the probable volume across a sampled population for a particular neuroanatomy in the template space. Their exact similarity and differences from probabilistic tractography at a group level should be explored in future studies. Additionally, the fidelity of the merged anatomy is often higher than what would be available from an individual. The result supports the validity of this technical approach. It is possible that the resulting average delineations can be used to generate population-specific anatomy atlases. It is also possible to use fiber density images rather than binary mask for conjunction. However, the result of an average fiber density image is difficult to interpret, and therefore should be explored in future studies.

When attempting to judge tractography delineation quality, human experts often are able to assess a particular delineation by visually identifying known anatomical priors. Direct tractography visualizations suggest that wide streamline dispersions often obscure anatomical details in visualizations, making anatomical assessment difficult (**Figure 4**). Therefore, image conjunctions offer better ability to assess reproducibility than direct tractography renderings alone. We aimed to develop an automated assessment of tractography reproducibility, since (a) researchers are often prone to judgment bias when exposed to large number of tractography results, and that subtle differences in results are often hard to distinguish, and (b) in an automated pipeline, the assessment score is necessary to allow iteratively fine tuning of tractography parameters. Since current neuroimaging techniques are not yet able to reliably classify neuroanatomy from tractography or image-based morphology, it is desirable for a computing pipeline to optimize and present the most reproducible tractography delineating across a population based on a particular set of ROIs as a first step. Subsequent anatomical assessment can then be made by a human expert, and the appropriate changes in ROI filter strategy can be made. This approach allows researchers with strong neuroanatomy backgrounds, but are less technically inclined, to make better judgments when performing group tractography.

The NOS and rater scores correlated significantly in filtered tractography. There was, however, little correlation in the case when tractography were unfiltered. The NOSs are closely related to the change in visual scale of the averaged result; thus, suggesting that human ratings assessments of filtered tractography are well encapsulated by the NOS, whereas the human decision factors for unfiltered tractography are more complex. Since the rating questionnaire is based on yes/no decisions on subanatomy identification, it is possible that the raters preferred to err more on false-positives than risking false-negatives, and this is what contributes to the differences in unfiltered tractography ratings. This means that human rating can become unpredictably biased with different filtering parameters and a more stable rating metric such as NOS may be more desirable.

For the specific tractography algorithms, CST*_prob_* produces the highest ratings, and performs particularly well in the optical radiation. It also results in more false-positives. This is most evident in CN VII/VIII and CN X results. In these cases the cranial nerve anatomies are very specific and local, and yet CST*_prob_* produces more false-positive projections to distant regions. In the fornix there are also erroneous tract extensions into the corpus callosum. Deterministic methods in comparison are more conservative and therefore result in lower scores. They perform well when the anatomy is regional and has finer features. Both CST*_det_* and XST performed well with cranial nerves. XST is able to image the rubrocerebellar pontine decussation with more reliability than CST*_det_*. While CST*_prob_* decussation delineation shows the highest reproducibility, it also produces wider projection coverage that is harder to interpret. The low DTT score is not surprising, as the single-tensor’s inability to resolve crossing-fibers is well known. Based on the result of the study, we recommend the use of CST*_prob_* for tasks in which the target anatomy is well defined and false positively can be easily recognized, or when interhemispheric and long projection distances are desired. For exploratory tractography, where filtering locations are not well defined, or when the anatomy features are close together, deterministic methods are recommended. When comparing CST*_det_* and XST, it appears that XST is more conservative and thereby results in less false-positives. It also has better ability in resolving hemispherical decussations, as evident by the results of the rubrocerebellar projections. In practice, the two methods are very close in performance. Note, however, that the comparison is not exhaustive of the possible parameter combinations on either method, and therefore automated tuning of the parameters can answer this question more definitively.

It is evident from this study that there is no single tractography algorithm that is superior in all aspects. The recent Tractometer challenge (Neher et al., 2015), which compared a wide range of tractography algorithms, including DTT and CST on a high resolution DWI phantom, has come to the same observation. It is clear that algorithm choice is highly task-dependent. Given that vast number of neuropathologies are not well understood, there is often no available ground truth of neuroanatomical measure in the clinical environment from which to form priors regarding algorithm performance. Automatic tuning of tractography parameters in order to maximize anatomy and population-specific reproducibility, and thereby allowing task-specific tractography algorithm recommendations is a highly desirable future direction. The SAGIT platform and NOS are a first step toward this goal.

### Limitations

For this study the DWI images were acquired at 0.94 × 0.94 × 3 mm^3^ voxel resolution. The dataset was acquired for the purpose of cranial nerve visualization on a 3 T GE HDx MRI with eight-channels head coil, and is therefore incapable of less than 2.6 mm isovoxel DWI resolution at a clinically acceptable scanning time. For example, the average trigeminal nerve, one of the larger cranial nerves, has a diameter of about 2 mm, as of such we compromised on an anisotropic voxel resolution to gain in-plane resolution. We believe our findings are novel for the application of clinical tractography delineations in less than ideal conditions.

The limit of the NOS is closely tied to the performance of its associated tractography algorithm. An algorithm and its associated parameters may consistently produce the wrong result in all subjects and produce a high NOS, or it may produce no results, and result in a NOS of 0. Both of these cases are regular occurrences in single-subject tractography analysis. With merged tractography, it is easier to recognize such faults. This is because in practice, incorrect tract paths are often the result of imaging anomalies or algorithm limit, and are unstable across a population. This can be observed as a general trend in **Figure 3**, for example, where DTI delineation of the rubrocerebellar tract is clearly incorrect, and results in low NOS. Therefore, NOS is capable of characterizing the uncertainty as a result of low algorithmic performance. In the case of false positives that are highly consistent, SAGIT can help researchers make more informed judgment.

## Conclusion

In this study, the SAGIT platform was created as an automated group tractography software platform that incorporated existing and proven dMRI practices, in order for group-wise dMRI to be more accessible to researchers. The tractography results demonstrated reliable and consistent performance of SAGIT across multiple subjects and techniques. By deploying SAGIT on 42 subjects, we quantifiably demonstrated that merged tractography is able to demonstrate algorithmic differences at a group-level. CST*_prob_* is prone to false-positives, and thereby is suitable when the targeted anatomy is well known. CST*_det_* and XST are more conservative, but have more trouble resolving hemispherical decussation and distant crossing projections. The NOS shows significant correlation with rater score for filtered tractography. Therefore it may be used for automated ratings of group tract results. As no single algorithm seems to be suitable for all anatomical tasks, it would be useful to consider using a mix of algorithms for different anatomical segments. Finally, we have demonstrated that merged tractography is a promising group-wise tractography analysis approach.

## Author Contributions

DC is the first author responsible for the original research and write up. JZ, DH, BB, MW, and PH contributed to the on going research design, and write up. MH is the principal investigator.

## Conflict of Interest Statement

The authors declare that the research was conducted in the absence of any commercial or financial relationships that could be construed as a potential conflict of interest.

## Footnotes

1 https://github.com/sinkpoint/sagit
