# Supplementary material for: Merged Group Tractography Evaluation with Selective Automated Group Integrated Tractography
Source: Front Neuroanat. 2016 Oct 13;10:96. doi: 10.3389/fnana.2016.00096 (PMC5061742; doi:10.3389/fnana.2016.00096)
Supplement: Supplementary file 2 [file Data_Sheet_1.docx]

# Tractography ROI definitions

| **Anatomy** | **Starting** | **Inclusive** | **Exclusive** |
| --- | --- | --- | --- |
| Fornix | Fornix column |  |  |
| CN VII/VIII | Cisternal CN VII/VIII stem |  | Ipsilateral Cerebellar peduncles |
| CN X | Cisternal CN X stem |  | Ipsilateral cerebellum from FreeSurfer |
| Rubrocerebellar | Red nucleus | Contralateral cerebellar white matter from FreeSurfer | Ipsilateral cerebellar whitematter from FreeSurfer |
| Optical radiation | Lateral geniculate | Ipsilateral V1/white matter boundary from FreeSurfer | Brainstem from FreeSurfer |
| Auditory radiation | Medial geniculate | Ipsilateral Heschl’s Gyrus/white matter boundary from FreeSurfer | Brainstem and Optical radiation inclusive region from FreeSurfer |
