# Supplementary material for: Merged Group Tractography Evaluation with Selective Automated Group Integrated Tractography
Source: Front Neuroanat. 2016 Oct 13;10:96. doi: 10.3389/fnana.2016.00096 (PMC5061742; doi:10.3389/fnana.2016.00096)
Supplement: Supplementary file 3 [file Data_Sheet_2.docx]

# Global tractography parameters

| **Method** | **Parameters** |
| --- | --- |
| CST_prob_ | -algorithm iFOD2 -step 0.5 -angle 45 -num 500 -minlength 10 -cutoff 0.15 -initcutoff 0.2 -force |
| CST_det_ | -algorithm SD_STREAM -step 0.3 -angle 45 -rk4 -num 300 -minlength 5 -cutoff 0.15 -initcutoff 0.2 -force |
| XST | -stop aniso:ca2,0.15 frac:0.1 radius:0.8 minlen:5 -step 0.5 |
| DTT | --stoppingvalue 0.15 --stoppingcurvature 0.8 --minimumlength 5 --clthreshold 0.15 --integrationsteplength 0.5 --randomgrid --seedspacing 0.3 |

## Region specific parameters

### **Auditory radiation:**

| **Method** | **Parameters** |
| --- | --- |
| CST_prob_ | -angle 30 |
| CST_det_ | -step 0.5 -angle 30 -rk4 -num 300 -minlength 10 -cutoff 0.10 -initcutoff 0.15 |
| XST |  |
| DTT | --minimumlength 10 --integrationsteplength 1 |
