# Supplementary material for: Merged Group Tractography Evaluation with Selective Automated Group Integrated Tractography
Source: Front Neuroanat. 2016 Oct 13;10:96. doi: 10.3389/fnana.2016.00096 (PMC5061742; doi:10.3389/fnana.2016.00096)
Supplement: Supplementary file 4 [file Data_Sheet_3.docx]

Supplementary Material Data Sheet 3

The complete visual report can be found on our lab website:

<http://www.hodaielab.com/sagit.html>

To download the visual reports:

<https://drive.google.com/open?id=0B7Atu7vLUEYjOGxnZDFOTVRwVXM>
